# Creating arts and crafting positively predicts subjective wellbeing

**DOI:** 10.3389/fpubh.2024.1417997

**Published:** 2024-08-16

**Authors:** Helen Keyes, Sarah Gradidge, Suzanna Elizabeth Forwood, Nic Gibson, Annelie Harvey, Evelin Kis, Karen Mutsatsa, Rachel Ownsworth, Shyanne Roeloffs, Magdalena Zawisza

**Affiliations:** Applied Social Change Hub, School of Psychology and Sport Science, Anglia Ruskin University, Cambridge, United Kingdom

**Keywords:** wellbeing, arts and crafts, life satisfaction, happiness, worthwhile life

## Abstract

**Introduction:**

This study explored whether engagement with Creating Arts and Crafting (CAC) predicted subjective wellbeing and loneliness, above and beyond known sociodemographic predictors.

**Methods:**

Secondary data from 7,182 adults living in England from the Taking Part Survey (a 2019–2020 UK household survey of culture and sport participation) were analyzed. Hierarchical Linear regressions were used to explore the predictive effect of engagement with CAC on aspects of subjective wellbeing (anxiety, happiness, life satisfaction and a sense that life is worthwhile) and loneliness. Covariates included gender, Index of Multiple Deprivation (IMD), age group, health and employment status.

**Results:**

Engaging in CAC significantly predicted increased life satisfaction, a sense that life is worthwhile and happiness, above and beyond known sociodemographic predictors.

**Conclusion:**

Our study provides support for the wellbeing benefits of engagement with creating arts and crafting, and we suggest that this will be a useful tool at a public health level, noting that relative accessibility and affordability creating arts and crafting.

## 1 Introduction

Since the COVID-19 pandemic, mental health provisions are failing to meet the growing demand on their services from people in need (1). Wellbeing describes how positive an individual’s psychological state is, which encompasses both feeling well [positive emotions and life satisfaction; (2)] and doing well [a sense of fulfillment; (3)]. Positive wellbeing is associated with a multitude of beneficial outcomes, including higher educational achievement (4), better physical health outcomes (5), more positive physical health behaviors (6) and reduced mortality (7). Loneliness is a negative psychological experience as a consequence of a deficit in high quality social connection (8, 9), and is associated with lower quality of life (10), as well as negative health behaviors and outcomes, such as substance misuse, smoking, eating disorders, depression, stress and premature death (8, 9). Any interventions to combat the profound public health issues of low wellbeing and high loneliness could therefore have a significant impact.

One suggested tool to improve wellbeing and combat loneliness is engagement with Creating Arts and Crafting (CAC). CAC can refer to numerous activities, such as pottery, drawing, painting, knitting, sewing, and crochet. There is wide-scale public interest in CAC, with British TV programs, such as *The Great British Sewing Bee* and *All that Glitters* populating prime time television slots. Approximately 20% of the British population are estimated to engage in arts and crafts (11). The arts and crafts industry contributes about £3.4 billion to the UK economy (12) and is forecast to be worth $50.91 billion worldwide by 2024 (13). Given its popularity, the unique purpose of this paper is to address gaps in the literature and directly assess the benefits of engagement in CAC on subjective wellbeing and loneliness, above and beyond known sociodemographic variable predictors within the general population.

Engagement with CAC is not a new intervention to improve mental health and wellbeing in clinical populations [e.g., (14)]. Indeed, CAC has been found to help with mental health issues (14, 15), enhance subjective wellbeing (16–20) and reportedly reduce suicidal tendencies (21). Therefore, not only is engagement with CAC of public interest, but it may also bring profound wellbeing benefits. As such, it has potential to be an optimal wellbeing intervention. However, while a substantial body of work has demonstrated the positive outcomes of engaging with CAC, most studies have investigated therapeutic interventions in clinical populations, only explored specific types of crafts and/or utilized small sample sizes, often with a qualitative approach [e.g., (14, 22–28)]. Thus, the extent to which the broad spectrum of CAC activities quantitatively contributes to wellbeing and loneliness outcomes in the everyday lives of the general population is comparatively understudied, and is the focus of this paper.

Less studied is whether engagement with CAC might be associated with reduced loneliness. Mindfulness is known to be effective in reducing loneliness [e.g., (29)], and there are parallels between mindfulness and CAC; motivations to engage in creative activities, including crafting, often center around their use as a tool to disengage from unwanted thoughts and feelings and to direct and focus attention onto the task at hand (30). This has been applied in therapies that use arts to facilitate mindfulness (31, 32). Considering the parallels between mindfulness and people’s motivations to engage in creative tasks, we seek to investigate here whether engagement with CAC can be effective in reducing loneliness.

### 1.1 Research question, aims and hypothesis

The current study aims to address these gaps in the literature. To this end we utilize a dataset from the Taking Part survey, an annual survey conducted by the UK Department for Culture, Media and Sport, which assesses a sample of the general population’s engagement in cultural, digital and sporting activities. We aim to quantitatively investigate whether engagement with *general* (rather than specific) CAC acts as a protective factor to enhance subjective wellbeing and reduce loneliness in a general (rather than clinical) population in a large, representative sample.

The current study controls for sociodemographic variables that are already known to be linked to subjective wellbeing and loneliness outcomes (33): level of deprivation, gender, age group, health and employment status. For instance, lower wellbeing and greater loneliness have been linked to greater deprivation (34, 35), poorer health (36, 37), and unemployment (38, 39). Loneliness also increases with age (40–42), with subjective wellbeing showing a U-shaped curve in high income countries, rising from the 45–54 age range onwards (36). Studies on gender differences in wellbeing and loneliness are more mixed [e.g., (42–45)] and sometimes yield only small effect sizes [e.g., (44)]. We include these sociodemographic variables in our analyses to: (a) control for statistical relationships between these known sociodemographic predictors and wellbeing/loneliness, and (b) to assess the relationship between engagement with CAC and wellbeing/loneliness *above and beyond* these known sociodemographic predictors. The latter enables us to determine by how much engagement with CAC contributes to subjective wellbeing and loneliness, in comparison to known sociodemographic predictors and their effect sizes. This assessment of magnitude allows us to evaluate how beneficial engagement with CAC may be to wellbeing and loneliness in a real world setting.

Wellbeing is measured here through Subjective Wellbeing (SWB), which comprises the variables of happiness, anxiety, life satisfaction and a sense that life is worthwhile, in line with the UK Office for National Statistics [ONS; e.g., (46)], as these variables provide a good measure of both feeling well [positive emotions and life satisfaction; (2)] and doing well [a sense of fulfillment; (3)]. The ONS has previously outlined the rationale for using these questions to measure subjective wellbeing (47). Our key research question asks: Does engagement with CAC predict SWB and loneliness *above and beyond* sociodemographic predictors of gender, deprivation, age group, health and employment? We predict that engagement with creating arts and crafting (vs. no engagement) will be associated with greater SWB and lower loneliness, over and above known sociodemographic predictors.

## 2 Methods

### 2.1 Participants

A random representative sample of 7,182 individuals were derived from the Taking Part Survey, a face-to-face household survey undertaken prior to COVID, in Year 13 (April 2019–March 2020), by the Department of Digital, Culture, Media and Sport (48). This dataset is available from the UK Data Service (48). The participants were aged 16 and over and living in England. Although the original dataset has 7,502 participants, the final sample size used in the main analyses for the current study was 7,182 due to missing data on outcome variables and/or covariates from 320 participants. Table 1 reports participants’ characteristics.

**Table 1:** Participant characteristics.

Characteristic	*N* (%) (Total *N* = 7,182)
Gender, *N* (%)	
Female	3,902 (54.3%)
Male	3,280 (45.7%)
Age group, *N* (%)	
Not reported	36 (0.5%)
16–19	21 (0.3%)
20–24	172 (2.4%)
25–34	925 (12.9%)
35–44	1,229 (17.1%)
45–54	1,291 (18%)
55–64	1,285 (17.9%)
65–74	1,223 (17%)
75–84	756 (10.5%)
85+	244 (3.4%)
IMD decile (scale = 1–10), *N* (%)	
1 (relatively most deprived decile)	732 (10.2%)
2	722 (10.1%)
3	613 (8.5%)
4	650 (9.1%)
5	772 (10.7%)
6	707 (9.8%)
7	770 (10.7%)
8	775 (10.8%)
9	778 (10.8%)
10 (relatively least deprived decile)	663 (9.2%)
Self-reported poor health (scale = 1–5), mean (SE)	2.11 (0.012)
In employment	
Yes, *N* (%)	3,995 (55.6%)
No, *N* (%)	3,187 (44.4%)
Engagement with creating arts and crafting	
Yes, *N* (%)	2,689 (37.4%)
No, *N* (%)	4,493 (62.6%)
Satisfaction (scale = 1–10), mean (SE)	7.76 (0.022)
Happy (scale = 1–10), mean (SE)	7.64 (0.025)
Worthwhile (scale = 1–10), mean (SE)	8.00 (0.021)
Anxiety (scale = 1–10), mean (SE)	2.79 (0.035)
Lonely (scale = 1–5), mean (SE)	2.22 (0.014)

Index of multiple deprivation (IMD), Number (N), Standard error (SE).

### 2.2 Measures

#### 2.2.1 Sociodemographics

As per Table 1, participants reported sociodemographic data regarding their age (categorical), gender (binary: *male* vs. *female*) and home postcode. The latter informed an index of multiple deprivation (IMD (49, 50)). IMD ranges from 1 to 10 where 1 represents the relatively most and 10 least deprived deciles, respectively. Participants were also asked to self-report their health (‘*How is your health in general?*’, 1–5, higher scores indicate poor health) and employment status (‘*Are you working?*’, options: *‘working’* vs. ‘*not working*’).

#### 2.2.2 Engagement with creating arts and crafting

To capture this concept, we compared participants who indicated they engaged in at least one of the following activities within the last 12 months (vs. none): ‘*Painting, drawing, printmaking or sculpture*’ (question artp13), ‘*Photography as an artistic activity (no family or holiday ‘snaps’)*’ (artp14), ‘*Made films or videos as an artistic activity (not family or holidays)*’ (artp15), ‘*Used a computer to create original artworks or animation*’ (artp16), ‘*Textile crafts such as embroidery, crocheting or knitting*’ (artp17), ‘*Wood crafts such as wood turning, carving or furniture making*’ (artp18) and ‘*Other crafts such as calligraphy, pottery or jewellery for yourself*’ (artp19). Specifically, we used the variable artp263Y13 (whereby participants were asked to indicate if they participated in ‘none of these’ activities) and reversed coded this variable for the current study, so that one indicated a participant had taken part in at least one craft activity, and zero indicated a participant had not taken part in any of these craft activities. Overall, 37.4% of participants in the survey had engaged in at least one craft activity within the past 12 months, while 62.6% had not.

#### 2.2.3 Subjective wellbeing (SWB)

Subjective wellbeing was measured through four single-item questions, whereby each was assessed on 0–10 Likert scales and analysed individually following ONS guidance (44). These items were: life satisfaction: “*Overall, how satisfied are you with your life nowadays*” (0 = “*not at all satisfied?*” to 10 = “*completely satisfied*”), life being worthwhile: “*To what extent do you feel that the things in your life are worthwhile?*” (0 = “*not at all worthwhile*” to 10 = “*completely*”), happiness: “*Taking all things together, how happy would you say you are?*” (0 = “*extremely unhappy*” to 10 = “*extremely happy*”), and anxiety: “*On a scale where 0 is “not at all anxious” and 10 is “completely anxious,” overall, how anxious did you feel yesterday?*” (0 = “not at all anxious” to 10 = “completely anxious”).

#### 2.2.4 Loneliness

A single item measured loneliness: “*How often do you feel lonely?*” where 1 = ‘*often or always*’, 2 = ‘*some of the time*’, 3 = *‘occasionally’*, 4 = ‘*hardly ever*’ and 5 = *‘never’*. This variable was reverse-coded for the current study, so that higher scores reflect greater loneliness.

## 3 Results

### 3.1 Analyses

To test our hypothesis, we ran hierarchical linear regressions, with engagement with CAC and sociodemographics (gender, level of deprivation, age, general health and employment status) as the predictor variables, and SWB (life satisfaction, life being worthwhile, happiness and anxiety) and loneliness as the outcome variables. Therefore, a total of five hierarchical regressions were conducted, one on each outcome variable. Sociodemographic variables were entered in Block 1, with engagement with CAC entered in Block 2. All analyses were conducted via Jamovi. The categorical predictor variables were dummy coded: engagement with CAC (reference: none of these), gender (reference: female), and employment status (reference: not working). There was no multicollinearity between the predictor variables, as assessed through VIFs ≤1.37.

#### 3.1.1 Life satisfaction

Engagement with creating arts and crafting (CAC) significantly predicted participants’ reported life satisfaction, above and beyond the effects of age, gender, deprivation, poor health, and being in work. Inclusion of engagement with CAC in the model explained an additional 0.1% of variance in life satisfaction scores compared to the effects of a model including only age, gender, deprivation, poor health and being in work, *F* (1, 7,175) = 4.59, *p* = 0.032. Engagement with CAC predicted a greater increase in life satisfaction (*β* = 0.088) than the increase associated with living in a less deprived area (*β* = 0.018). In the final stage of the model, deprivation, age, poor health and engaging with CAC were all significant predictors of life satisfaction, with those in less deprived areas, older age groups, those in better health and those who had engaged in CAC in the last year all reporting higher life satisfaction scores. The final model accounted for 16.4% of the variance in life satisfaction scores, *R*^2^ = 0.164, *F* (6, 7,175) = 235, *p* < 0.001. These findings align with our hypothesis.

#### 3.1.2 Sense of life being worthwhile

Engagement with CAC significantly predicted participants’ sense that life is worthwhile, above and beyond the effects of age, gender, deprivation, poor health and being in work. Indeed, inclusion of engagement in CAC in the model explains an additional 0.4% variance in participants’ sense that life is worthwhile compared to a model that only includes age, gender, deprivation, poor health and being in work, *F* (1, 7,175) = 27.5, *p* < 0.001. Participants’ engagement with CAC in the last 12 months had a larger effect on their sense that life is worthwhile (*β* = 0.218) than being in employment (*β* = 0.136), than aging by one decile (~ 20 years; *β* = 0.082) or than living in a less deprived area (*β* = 0.019). In the final model, all six variables significantly predicted participants’ sense that life is worthwhile, with women, those in older age groups, those living in less derived areas, those reporting better health, those in employment and those who had engaged with CAC in the last 12 months all reporting a greater sense that life is worthwhile. The final model accounted for 11.2% of variance in scores measuring a sense that life is worthwhile, *R*^2^ = 0.112, *F* (6, 7,175) = 150, *p* < 0.001. This is in line with our hypothesis.

#### 3.1.3 Happiness

Participants’ reported happiness was significantly predicted by their engagement with CAC, above and beyond the effects of age, gender, deprivation, poor health and being in work. An additional 0.1% of variance in participants’ happiness scores could be explained by the inclusion of engagement with CAC in the model, compared to the effects of age, gender, deprivation, poor health or being in work, *F* (1, 7,175) = 6.71, *p* = 0.010. Engagement with CAC predicted a similar increase in happiness (*β* = 0.128) as aging by one decile (~ 20 years; *β* = 0.127). In the final stage of the model, gender, age, poor health and engagement with CAC all significantly predicted happiness scores, with women, those from an older age group, those reporting better health and those who had engaged with CAC in the last 12 months all reporting being happier. The final model accounted for 10.6% of variance in participants’ happiness scores, *R*^2^ = 0.106, *F* (6, 7,175) = 142, *p* < 0.001. These findings align with our hypothesis.

#### 3.1.4 Anxiety

Contrary to our hypothesis, engagement with CAC did not provide additional predictive power for anxiety scores to the model (*p* = 0.108). The final model accounts for 7.1% variance in anxiety scores, with women, those in younger age groups, and those in poorer health reporting higher anxiety scores, *R*^2^ = 0.071, *F* (6, 7,175) = 91.5, *p* < 0.001.

#### 3.1.5 Loneliness

Contrary to our hypothesis, a model including engagement with CAC did not predict significantly more variance in loneliness scores than a model including age, gender, deprivation, poor health and being in work alone (*p* = 0.805). The final model accounted for 7.7% of variance in loneliness scores, with men, older age groups, those living in less deprived areas, those in better health and those in employment all reporting being less lonely, *R*^2^ = 0.077, *F* (6, 7,175) = 99, *p* < 0.001.

### 3.2 Summary

Our findings support aspects of our hypothesis; specifically that engagement with CAC is associated with significantly higher life satisfaction, happiness, and a sense that life is worthwhile. Engagement with CAC is not associated with lower levels of anxiety or loneliness (Table 2).

**Table 2:** Results of two-step hierarchical multiple regressions predicting satisfaction, happiness, life being worthwhile, anxiety and loneliness with regression coefficients (B) specified for all predictor variables at each step of the regression.

Predictor variables	Satisfaction	Happiness	Worthwhile	Anxiety	Loneliness
Step 1					
Intercept	8.694***	8.418***	8.682***	2.559***	2.183***
Gender	−0.071	−0.145**	−0.234***	−0.387***	−0.209***
IMD decile	0.019**	0.012	0.021**	0.021	−0.019***
Age group	0.100***	0.126***	0.082***	−0.239***	−0.048***
General health	−0.749***	−0.697***	−0.571***	0.753***	0.284***
In employment	0.062	0.019	0.136**	0.002	−0.187***
*R* ^2^	0.163	0.105	0.108	0.071	0.077
*F*	280***	169***	174***	109.3***	118.8***
Step 2					
Intercept	8.653***	8.358***	8.580***	2.506***	2.187***
Gender	−0.063	−0.128**	−0.205***	−0.371***	−0.210***
IMD decile	0.018*	0.011	0.019**	0.020	−0.019***
Age group	0.100***	0.127***	0.083***	−0.238***	−0.048***
General health	−0.747***	−0.695***	−0.567***	0.755***	0.284***
In employment	0.062	0.018	0.136**	0.002	−0.187***
ECAC	0.088*	0.128*	0.218***	0.113	−0.007
*R* ^2^	0.164	0.106	0.112	0.071	0.077
Δ*R*^2^	0.0005	0.0008	0.003	0.0003	<0.0001
Δ*F*	4.59*	6.71*	27.5***	2.59	0.061

*p < 0.05; **p < 0.01, ***p < 0.001.

## 4 Discussion

Given the concern for improving the population’s wellbeing (1) and decreasing loneliness (51), this paper set out to uniquely and directly assess the benefits of engagement with CAC to combat poor wellbeing and loneliness. For the first time, our paper demonstrates that engaging in general (rather than specific) crafting and arts activities may be beneficial in improving subjective wellbeing, over and above one’s gender, health, age, employment status and affluence. Importantly, we show that this pattern holds for the general (rather than clinical) population using quantitative (rather than qualitative) methods, addressing several gaps in the literature.

Specifically, in line with our hypothesis, engagement with CAC was linked to significantly increased life satisfaction, a greater sense that life is worthwhile and increased happiness, above and beyond known sociodemographic predictors. While these findings are of a small-sized magnitude, they are comparable to the effects of known sociodemographic variables, and are of practical importance at a population level. For example, the predictive effect of engagement with CAC on life satisfaction that we observed is an order of magnitude greater than the effect of living in a less deprived area. Similarly, the predictive effect of engagement with CAC on people’s sense that life is worthwhile is greater than the individual effect of being in employment, greater than the effect of living in a less deprived area, and greater than the effect of aging by one decile (~ 20 years). The effect of engagement with CAC on happiness was of a similar magnitude as the effect of aging by one decile or of being female. That is, although variances explained by engaging with CAC were objectively low, they are relatively larger (for life satisfaction and life being worthwhile) or comparable (for happiness) than those explained by other sociodemographic variables known to impact wellbeing. The relationships between engagement with CAC and subjective wellbeing measures in relation to sociodemographic predictors is noteworthy, because unlike static sociodemographic variables, engagement with CAC is a variable that can be manipulated and is therefore open to intervention. Thus, not only is engagement with CAC possibly more effective than many sociodemographic variables on improving aspects of wellbeing, but it is also an easier variable to manipulate and influence. We note also that engagement with crafting or creating arts has a relatively low entry point; crafting and creating arts is an accessible and relatively affordable pastime, and a popular pursuit among the general population. This is in contrast to attending live sporting events, which has also been demonstrated to positively predict SWM in a large UK sample (33), though attending live sporting events predicted lower loneliness, whereas engagement with CAC did not.

The implications of these findings are particularly significant given the importance of wellbeing. Higher SWB predicts health (52) and longevity (53), with some evidence pointing to positive effects on those with certain illnesses (54). Diener and Chan (55) outline the causal evidence demonstrating the beneficial influence SWB has on health and longevity. They also suggest policy makers include interventions to improve SWB across the population, given the potential impact of small SWB increases. Engagement with CAC is related to three of the four SWB measures assessed in this study. So, while the effects may be small, the contribution to SWB as measured by life satisfaction, happiness and life being worthwhile may provide a meaningful influence across society as a whole.

Our hypothesis was not supported with regards to one component of SWB (anxiety) and loneliness, as engagement with CAC had no significant predictive effect on either, above and beyond other sociodemographic predictors. Arts and crafts activities included here (e.g., drawing, painting, knitting, sewing, and crochet) can be considered as relatively solitary activities. This likely explains why we did not observe an effect of engaging with CAC on reducing loneliness. Further research should unpick the importance of the social elements of engagement with CAC, especially given the rich literature on social support and wellbeing (56).

Regarding the role of sociodemographic factors on SWB and loneliness, we largely replicate findings from previous research, as expected. We found that loneliness and perceiving life as worthwhile were both predicted by all sociodemographic variables here (deprivation, age group, gender, general health and employment status). Life satisfaction was predicted by deprivation, age and general health, whereas happiness and anxiety were both predicted by gender, age and health. In general, better health (36, 37) and older age (36, 40–42) are predictive of decreased loneliness and higher SWB. Further, higher levels of life satisfaction and the sense that life is worthwhile were associated with living in less deprived areas, as shown in previous research (34, 35). Similarly, loneliness is reported as lower in less deprived areas (57, 58). Previous literature also supports employment status being linked to factors associated with enhanced SWB (38, 59) and decreased loneliness (39). We found gender was not associated with life satisfaction, and previous literature has also been mixed in this regard (42–44, 57).

While our study’s strengths include the use of a nationally representative sample, investigation of overall engagement in craft activities and the use of quantitative methods, it is not without limitations. Firstly, our list of arts and crafts is not exhaustive and other arts categories, such as metalworking and literary arts were not considered. We also cannot be sure that participants shared the same understanding of what the various crafts listed in the survey entail. Of interest, many of the crafts included here may be seen as stereotypically feminine and thus the choice of CAC activities may be confounded with gender as a sociodemographic. It is unclear whether different arts and crafts affect men and women differently. Literature on masculinity threat and precarious manhood suggests that crafts such as knitting may be seen as threatening for many men (60). This question awaits further investigation, in particular whether stereotypically ‘masculine’ crafts (e.g., carpentry, metalworking) may have a larger impact on men’s wellbeing (29, 30). Secondly, it is important to note that the present study was correlational and therefore while we can speculate on the impact of engaging with CAC on life satisfaction, causation cannot be determined. The next step for future research is to experimentally manipulate whether or not participants engage in CAC and for how long to measure the causal impact on life satisfaction.

Further studies should also focus on the mechanisms through which engagement with CAC relates to wellbeing, which were not measured here. For example, a key component of creativity is the experience of flow (61), an intrinsically rewarding state characterized by total immersion in an activity. Frequent flow experiences have been linked to better quality of life, in particular wellbeing, satisfaction and a sense of mastery (62, 63). Creative arts are also associated with improved self-esteem and emotion regulation, and provide a means of authentic self-expression (64–70). A range of studies show that engagement in art therapy results in a reduction in anxiety (71), depression (72) and stress (73, 74), and improvements in social connection, wellbeing and life satisfaction (27, 68, 75, 76). Determining the exact mechanisms through which engagement with CAC may be beneficial for well-being awaits further studies.

Overall, our study provides support for exploring the wellbeing benefits of engagement with creative arts and crafting as a useful strategy to improve wellbeing at a population level, with the positive effects observed here being comparable to or greater than known sociodemographic predictors of wellbeing. Being already popular (37.4% of the current national sample), relatively cheap and accessible, engagement with CAC activities lends itself to government support and public uptake. Increased funding for creative arts and crafting activities for the general population may benefit society as a whole by improving wellbeing in modern living.

## Data availability statement

Publicly available datasets were analyzed in this study. This data can be found here: https://beta.ukdataservice.ac.uk/datacatalogue/doi/?id=8745#!#0.

## Ethics statement

Ethical approval was not required for the study involving humans in accordance with the local legislation and institutional requirements. Written informed consent to participate in this study was not required from the participants or the participants’ legal guardians/next of kin in accordance with the national legislation and the institutional requirements.

## Author contributions

HK: Conceptualization, Formal analysis, Investigation, Methodology, Writing – original draft, Writing – review & editing. SG: Conceptualization, Formal analysis, Investigation, Methodology, Writing – original draft, Writing – review & editing. SF: Conceptualization, Formal analysis, Investigation, Methodology, Writing – original draft. NG: Conceptualization, Formal analysis, Investigation, Methodology, Writing – original draft. AH: Writing – review & editing. EK: Conceptualization, Formal analysis, Investigation, Methodology, Writing – original draft, Writing – review & editing. KM: Writing – review & editing. RO: Conceptualization, Formal analysis, Investigation, Methodology, Writing – original draft, Writing – review & editing. SR: Conceptualization, Investigation, Writing – original draft. MZ: Conceptualization, Formal analysis, Investigation, Methodology, Project administration, Writing – original draft, Writing – review & editing.
